# Assessment of Placental Extracellular Vesicles-Associated Fas Ligand and TNF-Related Apoptosis-Inducing Ligand in Pregnancies Complicated by Early and Late Onset Preeclampsia

**DOI:** 10.3389/fphys.2021.708824

**Published:** 2021-07-23

**Authors:** Paola Ayala-Ramírez, Catalina Machuca-Acevedo, Tatiana Gámez, Sandra Quijano, Alfonso Barreto, Jaime L. Silva, Mercedes Olaya-C, Reggie García-Robles

**Affiliations:** ^1^Human Genetics Institute, Faculty of Medicine, Pontificia Universidad Javeriana, Bogotá, Colombia; ^2^Grupo de Inmunobiología y Biología Celular, Unidad de Investigación en Ciencias Biomédicas, Facultad de Ciencias, Pontificia Universidad Javeriana, Bogotá, Colombia; ^3^Department of Obstetrics and Gynecology, Faculty of Medicine, Hospital Universitario San Ignacio, Pontificia Universidad Javeriana, Bogotá, Colombia; ^4^Department of Pathology, Faculty of Medicine, Hospital Universitario San Ignacio, Pontificia Universidad Javeriana, Bogotá, Colombia; ^5^Department of Physiological Sciences, Faculty of Medicine, Pontificia Universidad Javeriana, Bogotá, Colombia

**Keywords:** pregnancy, hypertension disorder complicating pregnancy, exosome (vesicle), apoptosis, placental culture

## Abstract

Preeclampsia (PE) is a hypertensive disorder that affects 2–8% of pregnancies and is one of the main causes of fetal, neonatal, and maternal mortality and morbidity worldwide. Although PE etiology and pathophysiology remain unknown, there is evidence that the hyperactivation of maternal immunity cells against placental cells triggers trophoblast cell apoptosis and death. It has also been reported that placenta-derived extracellular vesicles (EV) carry Fas ligand (FasL) and Tumor necrosis factor-related apoptosis-inducing ligand (TRAIL) and trigger apoptosis in Jurkat T cells. This study aimed to quantify and compare FasL and TRAIL expression in EV derived from cultures of placenta explants from women with PE (early versus late) and women with uncomplicated pregnancies. Also, the study assessed EV capacity to induce apoptosis in Jurkat T cells. The authors isolated EV from placenta explant cultures, quantified FasL and TRAIL using ELISA, and analyzed EV apoptosis-inducing capability by flow cytometry. Results showed increased FasL and TRAIL in EV derived from placenta of women with PE, and increased EV apoptosis-inducing capability in Jurkat T cells. These results offer supporting evidence that EV FasL and TRAIL play a role in the pathophysiology of PE.

## Introduction

Preeclampsia (PE) is a hypertensive disorder of pregnancy with an impact on perinatal and maternal mortality and morbidity. According to the World Health Organization (WHO), maternal mortality due to such hypertensive disorders is 2.8% of pregnancies worldwide (Say et al., 2014). The mortality rate for PE in Latin America and the Caribbean is 26% (Steegers et al., 2010), and in Colombia 20–30% (Khan et al., 2006). Also, one fourth of fetal and neonatal deceases worldwide is associated with PE (500,000 neonates per year) (Duley, 2009).

This multifactorial disease is of unknown pathophysiology. The evidence, though, suggests the placenta plays an important role in the development of the disease. Increased proliferation and fusion of the cytotrophoblast, abnormal/incomplete remodeling of uterine spiral arteries (UtA), failed trophoblastic invasion, abnormal placentation, placental insufficiency, and an increased release of placenta-derived factors and EV have been described. These EV would have proinflammatory, anti-angiogenic, prothrombotic, and apoptotic properties. It is possible that placental molecules and membrane particles released in maternal circulation play a role in the systemic inflammation, endothelial dysfunction, and multi-systemic involvement characteristic of PE (Tannetta et al., 2017).

Currently, there are two categories of PE: Early-onset PE (EO-PE) (gestation < 34 weeks), and late-onset PE (LO-PE) (gestation ≥ 34 weeks). The two sub-types seem to have different etiology, pathophysiology, phenotype, and prognosis (Gilani et al., 2016). The EO-PE apparently involves an abnormal placentation and incomplete/failed remodeling of UtA. This type of PE has a higher risk of maternal and fetal complications such as intrauterine growth restriction (IUGR). The LO-PE possibly arises from the interaction between an apparently normal placenta and a maternal susceptibility involving endothelial dysfunction (microvascular damage) that results in general vasoconstriction and reduction of blood flow to multiple organs (heart, kidney, brain). This type of PE has a lower rate of fetal involvement and fewer perinatal complications than EO-PE (Phipps et al., 2016).

One of the principal mechanisms involved in PE development is apoptosis, associated with reduced/altered syncitiothrofoblast formation, abnormal UtA remodeling, increased proinflammatory cytokines, reduced maternal-fetal immune tolerance, and increased expression of apoptotic proteins such as FasL and TRAIL in placenta-released EV (Sharp et al., 2010). TRAIL is a type II membrane protein. This protein induces apoptosis in transformed cells and tumor cells, is expressed at a significant level in most normal tissues and has been implicated in processes of homeostasis, autoimmune suppression, immune surveillance, among others (Stelzer et al., 2016). FasL is a type II membrane protein that triggers death signals by binding to its membrane receptor Fas (Stenqvist et al., 2013). This protein is involved in the development of organs and in the homeostasis of cells and tissues. It is expressed in activated and cytotoxic T lymphocytes, NK cells, neutrophils, and cells found in immune privileged sites such as the eye, brain, testes, and placenta (Stelzer et al., 2016). It has been reported that FasL and TRAIL are expressed by trophoblast cells, which can induce apoptosis in activated lymphocytes and, therefore, provide a mechanism for the maternal immune privilege of the fetus, in addition to a regulation of placental homeostasis during trophoblastic invasion (Jerzak and Bischof, 2002). Different studies have shown that FasL is involved in the remodeling of the UtA and in the establishment of placental tolerance and immune privilege (Bai et al., 2009), for its part, the participation of TRAIL in the remodeling of UtA, where Cytotrophoblast uses a TRAIL-dependent mechanism to induce smooth muscle cell death and is involved in vessel remodeling (Keogh et al., 2007).

Our hypothesis was: there are differences between the amount of FasL and TRAIL in extracellular vesicles derived from culture of placental explants of pregnant women with uncomplicated pregnancy and pregnant women with diagnosis of EO-PE or LO-PE and these EVs are capable to induce apoptosis. This study aimed to assess the presence of FasL and TRAIL in EV released by the placentas of patients with EO-PE and LO-PE, and the apoptosis-inducing capability of those vesicle-associated proteins.

## Materials and Methods

### Sample Collection

This study was conducted in the Hospital Universitario San Ignacio (HUSI) from February to December, 2018. The samples were placentas of pregnant women with PE after cesarean section delivery (cases, *n* = 14). The study divided cases in 2 groups of 7 samples each: EO-PE (placentas from women diagnosed with PE that started at <34 week gestation) and LO-PE (placentas from women diagnosed with PE that started at ≥34 week gestation). Convenience sampling was performed.

Preeclampsia was diagnosed as the American College of Obstetricians and Gynecologists (ACOG) (The American College of Obstetricians and Gynecologists, 2019) recommendations. Placentas of women with uncomplicated pregnancies and healthy newborns were included as controls (controls, *n* = 7). Women with diabetes, kidney disease, intrapartum infection, or pregnancy complications, such as gestational diabetes, chorioamnionitis, and premature rupture of membranes were excluded. The authors reviewed the clinical histories of candidates for the study to determine whether they fulfilled inclusion criteria. The authors then informed potential participants of the research project and obtained informed consent from those choosing to participate. The placentas taken from C-sections were stored in plastic containers at 4°C in the Pathology laboratory and processed within 12 h of collection. The Ethics Committee of the HUSI-Pontificia Universidad Javeriana Faculty of Medicine approved the project (FM-CIE-0410-17). All procedures were carried out according to the biosafety manual of the Human Genetics Institute and the HUSI.

### Human Chorionic Villous Xplants Cultures

Each placental cotyledon was isolated using sterile dissection. The fetal and decidual tissues were removed, and blood from the interstitial space was washed out with 0.9% saline solution. Small fragments (explants) of approximately 0.5 μm were cut out. Two 6-well polyethylene plates were used for each sample. Four explants were incubated for 24 h (Miller et al., 2005) in each well in 8 ml of fetal bovine serum-free DMEM media (Gibco BRL, Bethesda, MD, United States) at 37°C (5% CO_2_). Culture viability was assessed using metabolic reduction of 3-(4,5-dimethylthiazol-2-ilo)-2,5-dypheniltetrazole bromide (MTT) (Sigma-Aldrich, St Louis, MO). Evaluation of culture cell function was by ß-hCG measurement by ELISA (Thermo Fisher Scientific Inc., Waltham, MA, United States).

### EV Collection

After 24 h of incubation, the supernatant of explants culture was centrifuged at 4,000 × *g* for 20 min. The pellet was used for another study, and the supernatant was ultra-centrifuged at 100,000 × *g* for 100 min. The supernatant was discarded, and the pellet re-suspended in 8 ml of PBS 1X. After a second ultracentrifugation of the supernatant at 100,000 × *g* for 90 min at 4°C, the supernatant was discarded, and the pellet again re-suspended in 50 μL of PBS 1X. The pellet was stored in 1.5 ml sterile vials at -20°C (Bautista et al., 2015).

### EV Quantification and Characterization

#### Nanoparticles Tracking Analysis (NTA)

For the NTA, a 2 ml pool was performed for EV samples. The diluted samples were loaded into the assembled sample chamber of a NanoSight NS300. The EV were brought into focus using the thumbprint region as a reference, and 60-s video images were acquired and analyzed with NanoSight NTA 3.4 software. The values obtained represent the mean and standard deviation of two replicate isolations.

#### Western Blot

The authors used Bradford colorimetric method (BIORAD) for total EV protein quantification. First, the standard curve was prepared, and then the samples loaded. EV protein lysates were resolved on Tris-Glycine SDS-PAGE and transferred to polyvinylidene difluoride (BIORAD) membranes. Membranes were incubated overnight in primary antibody CD63 (Invitrogen anti-CD63 catalog #10628D, diluted 1:500 per manufacturer instructions) at 4°C, washed three times with 0.1% TBST, incubated with secondary antibody (HRP-conjugated goat anti-mouse, Thermo Scientific, Pierce, 1:10,000) for 1 h at room temperature, washed three times and detected with enhanced chemiluminescence (Invitrogen) on CL-XPosure Film (Thermo Scientific, Pierce). In addition, IgG1 antibody was used as isotype control (Catalog # 02-6502; Invitrogen).

#### EV Quantification by ELISA

The ELISA ExoQuant^TM^ Overall Exosome Capture and Quantification Assay Kit (BioVision, California) was used for EV quantification, following the manufacturer’s protocol.

### VE FasL and TRAIL Quantification

The EV FasL quantification used the ELISA *Fas Ligand (APTL) Human Kit* (Abcam, Cambridge, MA, United States). The EV TRAIL quantification used ELISA *Human TRAIL/TNFSF10 (Tumor Necrosis Factor Related Apoptosis Inducing Ligand) K*it (Elabscience, Houston, Texas, United States). Manufacturer’s instructions were followed in both cases.

### Apoptosis Induction

The authors cultured 400,000 Jurkat T cells per well in 1 ml of RPMI-supplemented medium in three 12-well polyethylene plates. A 5mg/ml and 10 mg/ml EV protein concentration of 4 EV-sample pool from controls and from a 4 EV-sample pool from cases were added to Jurkat T cells. The samples were incubated at 37°C (5% CO_2_) for 8 and 24 h. Each reading assessed negative controls (Jurkat T cells with no EV exposure) and positive controls (Jurkat T cells + 5% DMSO) (Hajighasemi and Tajik, 2017). The FlowTACS^TM^ Apoptosis Detection Kit (Trevigen, Gaithersburg, MD, United States) was used to detect DNA fragmentation due to the apoptotic signaling cascade by flow cytometry in the BD FACSAria^TM^ III sorter (BD Bioscience, San Jose, CA, United States), following the manufacturer’s protocol. BDTM CompBeads were used to adjust the fluorescence signals and voltage for each detector. BDTM Cytometer Setup & Tracking Beads (CST) were used for daily evaluation of flow cytometer performance, also following the manufacturer’s recommendations.

### Statistical Analysis

The authors used the Mann-Whitney statistical test for a comparative analysis of demographic variables among groups. The Fisher exact test was used to compare categorical variables. The Mann-Whitney statistical test was also used to analyze differences in quantification of EV, EV total proteins, and EV FasL and TRAIL expression levels among groups. The tests were assessed with a 95% confidence level. Finally, flow cytometry was analyzed with the *FlowJo* v8 software, and a descriptive analysis was made of apoptosis percentage for each treatment.

## Results

There were statistically differences among cases and controls in newborn weight, gestational age at delivery, and blood pressure. Birth weight and gestational age at delivery were lower in cases, especially in EO-PE, compared to controls. The ratio of newborn weight/placenta weight was lower in EO-PE, compared to controls. As expected, gestational age at delivery was higher in LO-PE women compared to EO-PE women. Of the 14 pregnant women with PE, 8 (57.1%) presented PE severity criteria (blood pressure ≥ 160 mm Hg/ ≥ 110 mm Hg). In addition, 11 (78.5%) PE pregnant women had proteinuria as measured by urine protein/creatinine ratio (Table 1).

**TABLE 1:** Clinical and demographic characteristics of the population.

Variables			Cases						
	Controls (*n* = 7)		EO-PE (*n* = 7)			LO-PE (*n* = 7)			
	Median	Range	Median	Range	p value	Median	Range	p value	EO-PE vs. LO-PE p value
Maternal age (years)	31	26 – 39	34	23 – 42	0,9283	27	17 – 37	0,1719	0,1731
Gestational age at diagnostic (PE)	N/A	N/A	32,5	24,0 – 33,5	N/A	35,3	34,5 – 38,3	N/A	**0,0012**
Diastolic Blood pressure (mmHg)	62	58 –88	112	76 – 118	**0.008**	92	76 – 131	**0,01**	0,2554
Systolic Blood pressure (mmHg)	123	90 – 130	168,5	158 – 180	**0,001**	164,5	136 – 184	**0,001**	0,6753
Birth Weight (g)	3380	2800 – 3780	2287	1380 – 2590	**0,0006**	2640	2245 – 2950	**0,004**	**0,0175**
Gestational Age (weeks)	39	38 – 40	35	31 –36	**0,0006**	37	34 – 38	**0,004**	**0,0105**
Placental Weight (g)	403,9	328,9 – 439,2	397,7	138,0 – 430,0	0,3759	367,6	301,7 – 443,1	0,5221	0,9656
Birth weight/plcenta ratio (g)	8,7	6,7–9,9	6,42	5,3–10	**0,037**	7,1	6,4–8,0	0,097	0,097
Proteinuria/creatinuria ratio	N/A	N/A	0.9	0,2 – 14,7	N/A	0.55	0,3 – 18,2	N/A	N/A

	**N**	**%**	**n**	**%**	**p value**	**n**	**%**	**p value**	

**Primigravity**									
Yes	1	14,3	0	0	1,0	2	28,6	0,6	
No	6	85,7	7	100		5	71,4		
**Newborn sex**									
Female	2	28,6	4	57,1	0,5921	3	42,9	1,0	
Male	5	71,4	3	42,9		4	57,1		
**IUGR**									
Yes	0	0	1	14,3	1,0	1	14,3	1,0	
No	7	100	6	85,7		6	85,7		

*EO-PE, Early-onset preeclampsia; LO-PE, Late-onset preeclampsia; IUGR, Intrauterine growth restriction. Numbers in bold show p values <0.05.*

**FIGURE 1:**
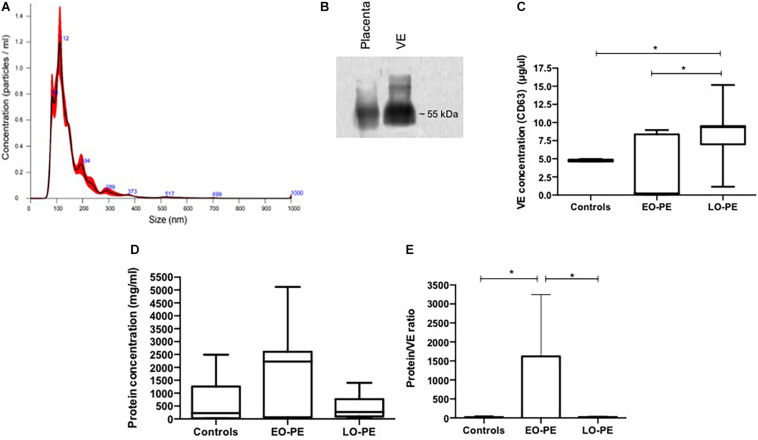
VE characterization from explant culture supernatant. **(A)** Representative vesicle size distribution (nm) by NTA of VE. **(B)** Western blot analysis for the CD63 exosome marker. **(C)** Quantification of VE by ELISA measuring CD63 (controls vs. EO-PE *p* = 0,2069; controls vs. LO-PE *p* = 0,0262; EO-PE vs. LO-PE: *p* = 0,0175). **(D)** Quantification of protein within VE by Bradford method (controls vs. EO-PE *p* = 0,2069; controls vs. LO-PE *p* = 0,6031; EO-PE vs. LO-PE: *p* = 0,3129). **(E)** Analysis of ratio of protein quantity by VE (controls vs. EO-PE *p* = 0,0175; controls vs. LO-PE *p* = 0,3759; EO-PE vs. LO-PE: *p* = 0,0262) *p ≤ 0,05.

Results of the nanoparticle tracking analysis allowed determination of EV isolation (Dragovic et al., 2015). The EV had an average size of 149.4 nm with standard deviation of 83.5 nm and concentration of 8.93e^8^ ± 3.74e^7^ particles/ml. Western blot for CD63 showed a ∼55 kDa band indicating the presence of EV enriched with exosomes in the process of isolation (Zhang et al., 2019) (Figure 1). The EV quantification showed a lower amount in samples from EO-PE (median: 0.24 ug/ul) compared to controls (median: 4.75 ug/ul) and compared to LO-PE cases (median: 9.36 ug/ul). The total protein quantification showed no statistically significant differences among groups (control group median: 228.5 mg/ml; EO-PE group median: 2,223 mg/ml; LO-PE group median: 268.7 mg/ml). The proportion of total protein/EV was higher in the EO-PE (median: 1,639-fold) compared to controls (median: 7.88-fold) and compared to the LO-PE group (median: 24.27-fold) (Figures 1C,D,E).

**FIGURE 2:**
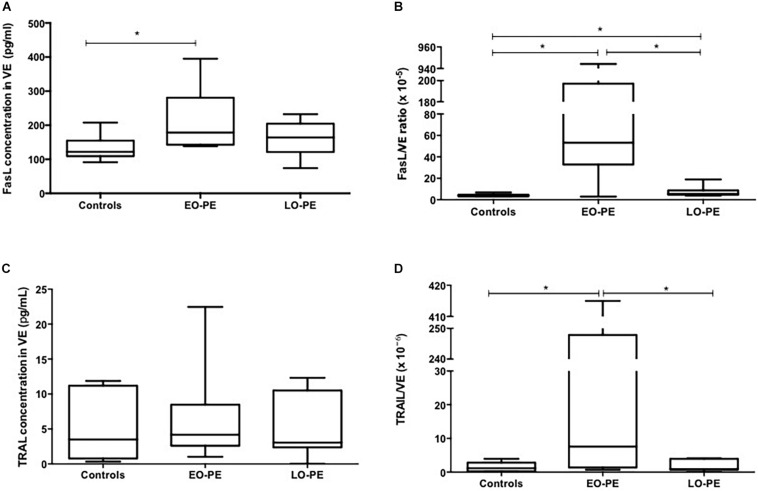
FasL and TRAIL quantification in VE. **(A)** Quantification of FasL in VE (controls vs. EO-PE *p* = 0,0379; controls vs. LO-PE *p* = 0,2564; EO-PE vs. LO-PE: *p* = 0,6031). **(B)** FasL/VE ratio (controls vs. EO-PE *p* = 0,0262; controls vs. LO-PE *p* = 0,0379; EO-PE vs. LO-PE: *p* = 0,0262). **(C)** Quantification of TRAIL in VE (controls vs. EO-PE *p* = 0,6894; controls vs. LO-PE *p* = 0,7791; EO-PE vs. LO-PE: *p* = 0,6894). **(D)** TRAIL/VE ratio (controls vs. EO-PE *p* = 0,0379; controls vs. LO-PE *p* = 0,9656; EO-PE vs. LO-PE: *p* = 0,0379). *p ≤ 0,05.

Regarding EV FasL expression, concentration in EO-PE (median 178.6 pg/ml) was higher compared to controls (median: 122 pg/ml). No differences were found between EO-PE and LO-PE (median 164.2 pg/ml) (Figure 2A). The FasL/EV proportion was higher in the EO-PE (median: 53.2 × 10^–5^-fold) compared to controls (median: 3.85 × 10^–5^-fold) and compared to the EO-PE group (median: 5.42 × 10^–5^-fold) (Figure 2B).

Results of EV TRAIL do not show statistically significant differences among groups (median for EO-PE: 4.17 pg/ml; for LO-PE: 3.05 pg/ml; for controls: 3.50 pg/ml) (Figure 2C). For the proportion of FasL concentration/EV concentration, the EO-PE (median: 7.57 × 10^–6^-fold) value was higher compared to the control group (median: 1.16 × 10^–6^-fold) and compared to the LO-PE group (median: 0.90 × 10^–6^-fold) (Figure 2D).

**FIGURE 3:**
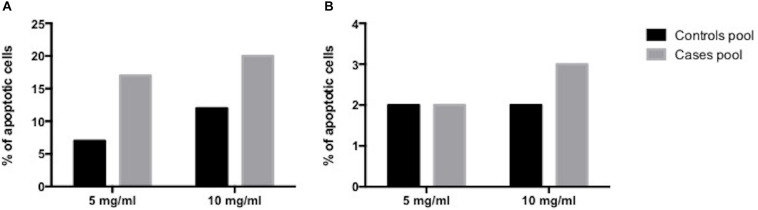
Human placenta-derived VE apoptosis induction analysis. **(A)** at 8 h **(B)** at 24 h.

Conversely, analysis of apoptosis in the EV-treated Jurkat T cells showed a directly proportional relationship between EV concentration increase and apoptosis percentage, at 8 and at 24 h. There was a reduction, however, in the apoptosis percentage at 24 h, both in cases and in controls (Figure 3 and Supplementary Figures 1, 2).

## Discussion

Pregnant women with EO-PE had less favorable obstetric results with high blood pressure levels (severity criterion), premature deliveries (gestation ≤ 36 weeks), and low birth weights, compared to controls and to LO-PE pregnant women. This is consistent with previous studies (Lok et al., 2008; Nguyen et al., 2019).

This study shows that placental explants from LO-PE pregnancies have a higher EV release *in vitro*. This phenomenon has been described by Dragovic *et al.*, who reported an increased release of syncytiotrophoblast-derived EV in LO-PE patients and controls compared to those in non-pregnant women (Dragovic et al., 2013). Several authors have found a significant increase of circulating exosomes in EO-PE women, possibly associated with abnormal placental development and reduced function (Lok et al., 2008; Redman et al., 2012; Pillay et al., 2016). Meanwhile, Marques *et al.* did not find significant differences in EV concentrations for PE patients and normotensive pregnant women (Marques et al., 2012). Discrepancy between these results may be due to differences in the types of analyzed samples and the methods for EV isolation and identification (Dragovic et al., 2013). Mitchell *et al.* propose that exosome release may reflect the placental function and metabolic status (Mitchell et al., 2015). It has been reported that in LO-PE the problem arises from interaction between a presumably normal placenta and maternal factors plagued with endothelial dysfunction, making them susceptible to microvascular damage (Phipps et al., 2016). The increased exosome release we report in LO-PE placentas could be interesting and need more research, this finding might be related to a compensation mechanism that attempts to mitigate a pregestational endothelial dysfunction. Further studies are necessary to prove that hypothesis. It is important to note that EV release has been reported to increase with gestational age (Salomon et al., 2014), so differences in EV amounts between EO-PE and LO-PE may be due to gestational age.

By contrast, assessment of the total proteins/EV proportion reveals an increase in the amount of total proteins per EV in EO-PE cases. This finding is consistent with some reports indicating an increased release of EV with altered molecular characteristics (mainly in the bioactive charge) during PE development. These altered characteristics may affect normal EV biological function (Tannetta et al., 2017). The EV express sFlt-1 and endoglin that may contribute to endothelial dysfunction. The EV also express plasminogen-activator inhibitors (PAI-1/PAI-2) responsible for the high levels of fibrin deposition in the intervillous space and for the placental infarctions observed in PE. The PAI are also responsible for excessive oxidation that may amplify the EV inflammatory burden modifying STB proteins and lipids that are not proinflammatory (Tannetta and Sargent, 2013).

Some studies have shown the effect of placental-derived exosomes in the maternal immune modulation during pregnancy, partly through the expression of pro-apoptotic molecules as FasL and TRAIL (Mitchell et al., 2015). FasL may induce the stimulation/activation of the vascular endothelium. FasL may also induce an altered trophoblastic apoptosis that would play a role in endothelial dysfunction, systemic inflammation, and hypertension (Gibbens et al., 2017). Conversely, it has also been reported that FasL may induce apoptosis in activated lymphocytes as a mechanism of tolerance and immunological privilege (Jerzak and Bischof, 2002). FasL also plays a role in the UtA remodeling (Bai et al., 2009). These mechanisms may be related to the EO-PE and the increased EV expression of FasL in EO-PE.

Regarding TRAIL, this study shows an increased TRAIL/EV proportion in the EO-PE group. This increase may be a response to STB reduction or functional loss caused by the altered apoptosis process during PE (Bai et al., 2009). It also may derive from the increased apoptosis induction in activated lymphocytes as a defense mechanism against fetal allograft rejection by the maternal immune system (Stenqvist et al., 2013).

The apoptosis-inducing capability of the isolated EV was assessed using the TUNEL assay. Placental EV has been reported to induce apoptosis in T cells as an immunological tolerance mechanism. For that reason, this experimental model (Sabapatha et al., 2006) used Jurkat T cells. Leukemic cells have been reported to express FasL on their surfaces, so they were also used in this model (Yamada et al., 2017). Previous *in vitro* studies have shown that EV from placenta and serum of healthy pregnant women induce a significant increase (3.38 fold) of Jurkat T cells apoptosis compared to EV isolated from the serum of non-pregnant women. Studies also show that apoptosis induction depends on the Fas/FasL complex (Gercel-Taylor et al., 2018). Gupta *et al.* in turn, showed that EV derived from explants cultures of placentas from uncomplicated pregnancies may play a role in the response of activated T cells (EV reduce T-lymphocyte proliferation and production of IL-2 and IFNγ). Those authors, however, did not find apoptosis induction in the cells (Gupta et al., 2005). Results of the present study show that the isolated EV are capable of triggering apoptosis in Jurkat T cells in a concentration-dependent manner (Stenqvist et al., 2013). There is also a higher apoptosis induction by EV from placentas of women with PE. This may result from EV triggering higher protection for the fetoplacental unit from activated maternal immunecells (Nair and Salomon, 2018). The higher apoptosis induction may also result from the inhibition of T lymphocyte activation and prolipheration (Gupta et al., 2005). A higher EV apoptosis-inducing capability may suggest EV content regulation aiming for higher apoptosis in target cells (Gilani et al., 2016). On the other hand, analysis of apoptosis with different treatments at 8 h and 24 h shows a reduction in apoptosis percentage at 24 h. *Zhang et al.*, working from studies reporting T lymphocyte phagocytic capacity, exposed Mycobacterium tuberculosis (H37Ra) to Jurkat T cells, noting some morphological changes in the Jurkat T cells (cytoskeleton remodeling, wrinkled cellular surfaces, pseudopodia formation) associated with the induction of a form of non-selective endocytosis named macropinocytosis (Zhang et al., 2015). The eventual phagocytic capacity of the Jurkat T cells may explain the reduction in the apoptotic cells percentage over time.

This is the first study to evaluate EV FasL and TRAIL levels from placentas with EO-PE and LO-PE and to assess their apoptosis-inducing capability. The principal limitation of this study is the sample size. It is recommended to replicate methods used in this study with a larger sample. Unfortunately, it is not possible to establish with certainty the nature of the isolated EV. Their size corresponds to exosomes, and analysis revealed the presence of CD63, a principal exosome marker (Zhang et al., 2019). This marker, however, has also been found in other EV, such as micro-particles (Rank et al., 2012) and apoptotic bodies (Crescitelli et al., 2013). The results in this study would have been complemented by measurement of FasL and TRAIL expression in placentas. A limited number of samples was a weakness that kept the apoptosis essays from reaching conclusions at a statistically significant level and from comparing EO-PE and LO-PE. Unfortunately, no replicas of the apotosis induction experiments were possible, due to the availability of extracellular vesicle samples, for this reason it is recommended to carry out new studies to replicate these results.

This study suggests that FasL and TRAIL molecules, present in EV and of placental origin, participate in the pathophysiology of EO-PE and LO-PE. Study results provide additional evidence of a possible role of EV in the immune tolerance regulation in normal pregnancy and in pathological conditions such as PE, probably also with participation of FasL and TRAIL. It is necessary to continue research on this subject to gain more knowledge. It will be important to precisely identify the immunological target cells and the molecular mechanisms involved. There is also a conundrum: Are other cell types, such as the maternal vascular endothelial cells, a target? If so, what is their possible role in PE?

## Data Availability Statement

The original contributions presented in the study are included in the article/Supplementary Material, further inquiries can be directed to the corresponding author/s.

## Ethics Statement

The studies involving human participants were reviewed and approved by the Ethics Committee of Hospital Universitario San Ignacio-Faculty of Medicine of Pontificia Universidad Javeriana of Bogotá, Colombia. The patients/participants provided their written informed consent to participate in this study.

## Author Contributions

PA-R and RG-R conceived, designed, planned, and supervised the experiments. SQ and AB supported the implementation of the apotosis and extracellular vesicle isolation experiment. CM-A and TG performed the experiments. MO-C and JS collected samples and data of patients. CM-A, PA-R, and RG-R processed and analyzed the data and drafted the manuscript. All authors provided critical feedback, contributed to the interpretation of the results, and approved the final manuscript.

## Conflict of Interest

The authors declare that the research was conducted in the absence of any commercial or financial relationships that could be construed as a potential conflict of interest. The handling editor declared a past co-authorship with the authors RG-R and PA-R.

## Publisher’s Note

All claims expressed in this article are solely those of the authors and do not necessarily represent those of their affiliated organizations, or those of the publisher, the editors and the reviewers. Any product that may be evaluated in this article, or claim that may be made by its manufacturer, is not guaranteed or endorsed by the publisher.

## Supplementary Material

Supplementary Figure 1 Human placenta-derived VE apoptosis induction plots at 8 h. **(A)** Selection of the population, **(B)** Elimination of duplets, **(C)** negative control, **(D)** positive control (DMSO), **(E)** VE control 5mg/mL, **(F)** VE control 10mg/mL, **(G)** VE pool of preeclampsia cases 5 mg/mL, **(H)** VE pool of preeclampsia cases 10 mg/mL.

Supplementary Figure 2 Human placenta-derived VE apoptosis induction plots at 24 h. **(A)** Selection of the population, **(B)** Elimination of duplets, **(C)** negative control, **(D)** positive control (DMSO), **(E)** VE control 5 mg/mL, **(F)** VE control 10 mg/mL, **(G)** VE pool of preeclampsia cases 5 mg/mL, **(H)** VE pool of preeclampsia cases 10 mg/mL.
