# Supplementary figures and images for: Assessment of Placental Extracellular Vesicles-Associated Fas Ligand and TNF-Related Apoptosis-Inducing Ligand in Pregnancies Complicated by Early and Late Onset Preeclampsia
Source: Front Physiol. 2021 Jul 23;12:708824. doi: 10.3389/fphys.2021.708824 (PMC8342945; doi:10.3389/fphys.2021.708824)

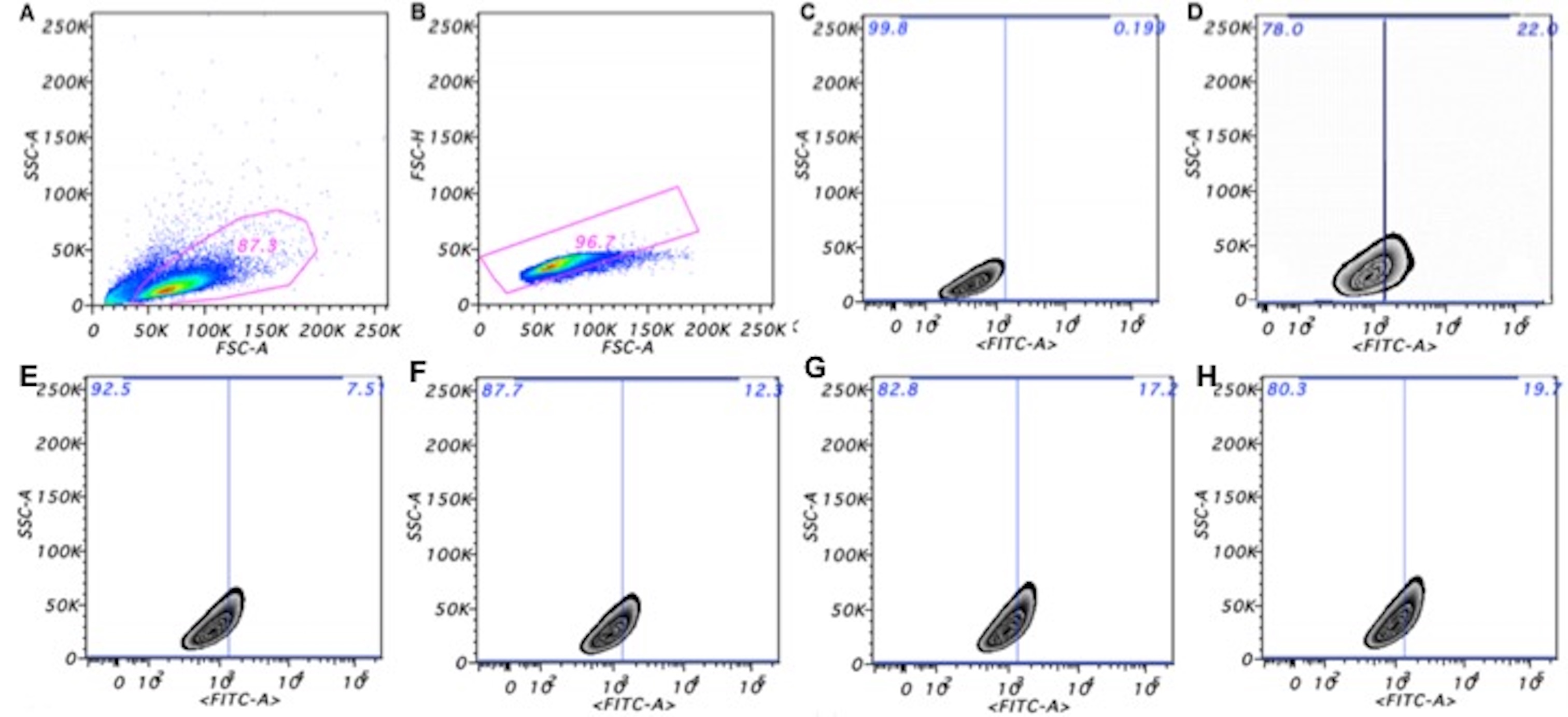

Supplement: Supplementary Figure 1 — Human placenta-derived VE apoptosis induction plots at 8 h. (A) Selection of the population, (B) Elimination of duplets, (C) negative control, (D) positive control (DMSO), (E) VE control 5mg/mL, (F) VE control 10mg/mL, (G) VE pool of preeclampsia cases 5 mg/mL, (H) VE pool of preeclampsia cases 10 mg/mL. [file Image_1.JPEG]

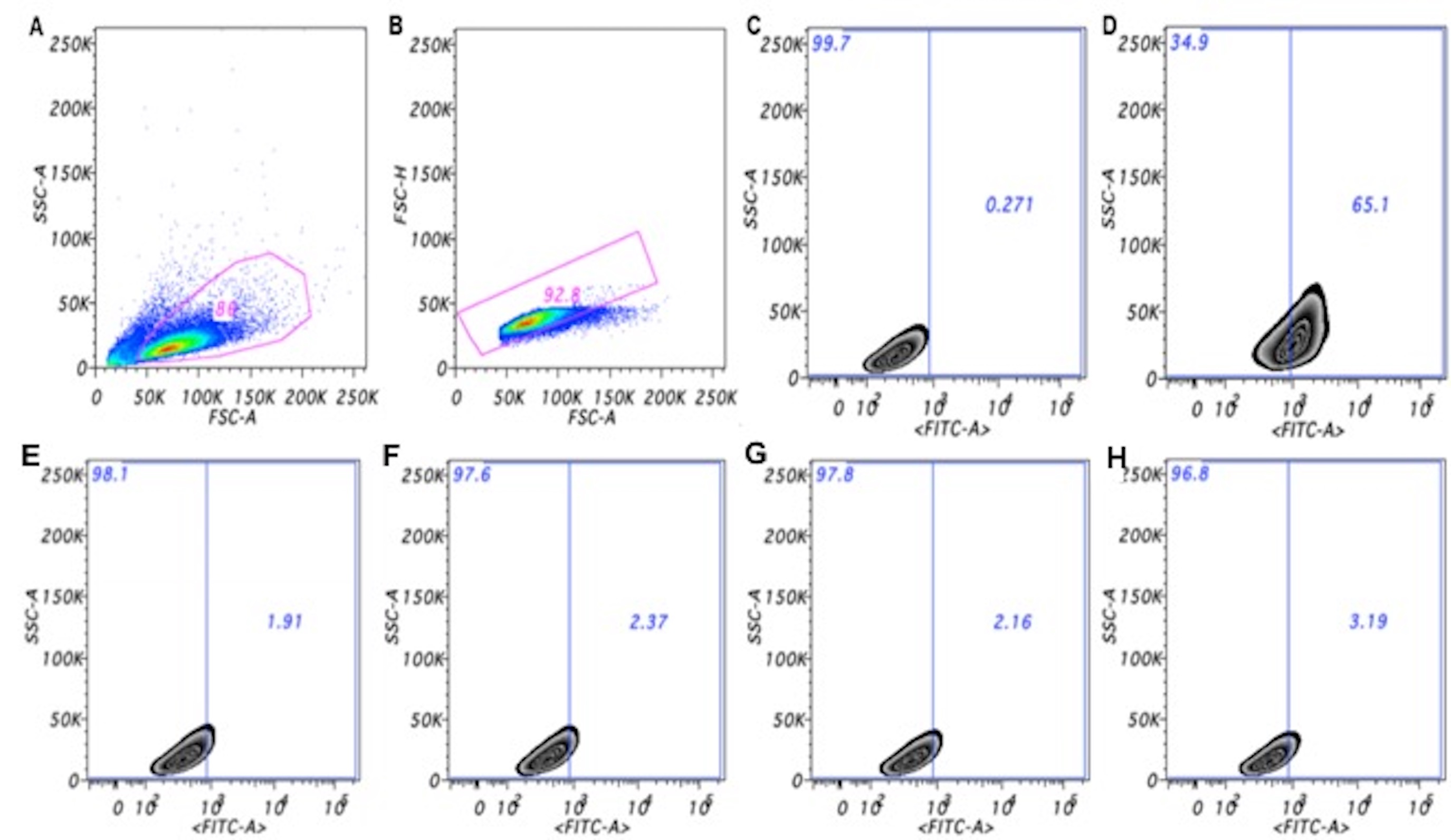

Supplement: Supplementary Figure 2 — Human placenta-derived VE apoptosis induction plots at 24 h. (A) Selection of the population, (B) Elimination of duplets, (C) negative control, (D) positive control (DMSO), (E) VE control 5 mg/mL, (F) VE control 10 mg/mL, (G) VE pool of preeclampsia cases 5 mg/mL, (H) VE pool of preeclampsia cases 10 mg/mL. [file Image_2.JPEG]
